# The exceptional abandonment of metal tools by North American hunter-gatherers, 3000 B.P.

**DOI:** 10.1038/s41598-019-42185-y

**Published:** 2019-04-08

**Authors:** Michelle R. Bebber, Alastair J. M. Key, Michael Fisch, Richard S. Meindl, Metin I. Eren

**Affiliations:** 1 Department of Anthropology, Kent State University, Kent, OH 44242 USA; 2 School of Anthropology and Conservation, University of Kent, Canterbury, CT2 7NZ UK; 3 College of Aeronautics and Engineering, Kent State University, Kent, OH 44242 USA; 4 Department of Archaeology, Cleveland Museum of Natural History, Cleveland, OH 44106 USA

## Abstract

Most prehistoric societies that experimented with copper as a tool raw material eventually abandoned stone as their primary medium for tool making. However, after thousands of years of experimentation with this metal, North American hunter-gatherers abandoned it and returned to the exclusive use of stone. Why? We experimentally confirmed that replica copper tools are inferior to stone ones when each is sourced in the same manner as their archaeological counterparts and subjected to identical tasks. Why, then, did copper consistently lead to more advanced metallurgy in most other areas of the world? We suggest that it was the unusual level of purity in the North American copper sourced by North American groups, and that naturally occurring alloys yielded sufficiently superior tools to encourage entry into the copper-bronze-iron continuum of tool manufacture in other parts of the world.

## Introduction

Metallurgy in North America may have begun as early as 7,000 years ago^1,2^. By the Middle and Late Archaic periods between 6000 and 3000 B.P. a florescence of copper working, known as the Old Copper Culture, thrived in and around the world’s largest naturally occurring pure copper deposit which is in North America’s Lake Superior region^3^. During these millennia, hunter-gatherers stretching from central Canada to the eastern Great Lakes regularly made utilitarian implements out of copper^4–12^, only for these items to decline in prominence and frequency as populations grew and social complexity increased during the Archaic to Woodland Transition^1,13–17^. After 3000 B.P. prehistoric people in Eastern North America continued to use copper, but it was mostly relegated toward ritualized items^16,18^.

Binford^19^ referred to this decline in utilitarian tools made from copper as the Old Copper Culture “technomic devolution”, and it is a unique event in archaeologists’ global understanding of prehistoric metallurgic evolution^20^. While the use of stone implements often continued into the metal ages^21^, analogous ones produced from metal ultimately replaced these implements. Indeed, the near-global transition from stone to metal tools during the early- and mid-Holocene appears to be a ubiquitous, unidirectional transition^22–26^. Cases where metal tools were indigenously innovated and used, but did not ultimately predominate or replace stone tools, are rare. Thus, the abandonment of Old Copper Culture utilitarian tools facilitates the examination of an exceptional situation in human prehistory: how and why metal tools were selected *against*.

Binford^19^ found this situation particularly “interesting” because of the general assumption that in terms of “absolute efficiency” copper tools were superior to their functional equivalents in stone, possessing both greater durability as well as superiority in accomplishing cutting and piercing tasks. However, acknowledging that the manufacture of copper tools would have required greater energy expenditure than stone tools, Binford^19^ maintained that copper tools would have still been more efficient in terms of net energy expenditure. This is because copper tools were “probably more durable and could have been utilized for a longer period of time”^19^. Thus, despite the greater energy required to produce a copper tool relative to a stone one, a copper tool’s durability would have conserved energy in task performance. Binford^19^ was less certain whether copper tools were superior to stone ones in cutting and piercing functions, suggesting that “only experiments can determine”^19^ that difference.

Current archaeological evidence is consistent with the hypothesis that population growth and increased social complexity contributed to the selection against utilitarian copper tools around 3000 B.P. Larger, more numerous, and more ostentatious cemeteries during the Late Archaic suggest that populations in the Upper Lakes were growing, and societies were becoming less egalitarian. One clear archaeological signal of increased burial ostentation is the interment of ornamental copper artifacts^13,15,19^. Thus, it has been argued that an increasingly socially complex world required an increase in ornamental copper production, resulting in a concomitant production decline in utilitarian copper tools^14–18,27^.

However, whether demographic and social factors *alone* led to the decline of utilitarian copper tools after 3000 B.P. is currently unknown because experimental tests examining Binford’s^19^ assumptions regarding copper versus stone tool durability and cutting ability have yet to be conducted. Here, we assess those assumptions with replicas of the implement best suited to test both of these factors simultaneously: knives. We use a mechanical engineering approach that measures the amount of energy expenditure needed to complete a simple task—cutting a uniform substrate—to evaluate whether or not there exist differences in durability and cutting ability between knives made from copper versus those made from stone.

## Materials and Methods

Thirty replica copper blades were produced by M.R.B.^20^. The specimens were suitable in shape for controlled materials testing, but similar in composition and internal structure to those produced during the Late Archaic^20^ (SI Appendix). The copper used for production of the experimental specimens was procured from same mining area that would have been used in ancient times, the Keweenaw Peninsula, Michigan^1,2,28^. Thirty stone flakes were produced by A.J.M.K. and M.I.E. from Keokuk chert, a common toolstone used throughout the North American Midwest. Each edge angle of a copper blade specimen corresponded to a similar edge angle of a stone flake specimen (SI Appendix).

Our sharpness and durability cutting experiments follow closely the procedures described in Key *et al*.^29^ (SI Appendix). We used an Instron Universal Materials Tester (Model 5967) in which peak force (N) and total work (J) during cutting were calculated for all specimens. Following Schuldt *et al*.^30^, force and work are used as proxies for edge sharpness. In lieu of biological tissues, modern mechanical tests of sharpness regularly employ flexible soft solid plastics as the cutting substrate^31–33^. This is due to the structural inconsistencies that exist in the muscle fibers of meat, which ultimately cause variation in the force and energy measurements. Here we use standard PVC (polyvinyl chloride) tubing with 6 mm O.D. cut to length of approximately 15 cm for mounting in the substrate grips.

We conducted three analyses comparing copper versus stone knives: initial sharpness, final sharpness, and durability. To assess initial sharpness, we measured the force and work necessary for the first cut of the substrate before the knives were blunted. To assess final sharpness, we averaged the force and work necessary to cut the substrate for each of the five subsequent cutting tests performed after a blunting event (SI Appendix). The lower force^30^ and work required for a cut indicated a sharper tool. To assess durability^34^ (in this case the ability of an edge to resist blunting over time), we used repeated test cuts with the same blade^35^ to examine how much more force and work was required to cut the substrate for the post-blunting cutting events versus the initial cut. A smaller difference between these two values indicated a more durable material.

The sharpness and durability data were analyzed using IBM SPSS version 23. The nonparametric Mann-Whitney *U* tests with Monte Carlo permutation (10,000 permutations) and 95% confidence intervals were used for the analyses. Mann-Whitney *U* is a conservative statistical procedure that requires only minimal assumptions of the data^36,37^. Effect size *r* was also calculated^37,38^. All raw data can be found in Dataset S1.

## Results

### Initial sharpness

**Figure 1:**
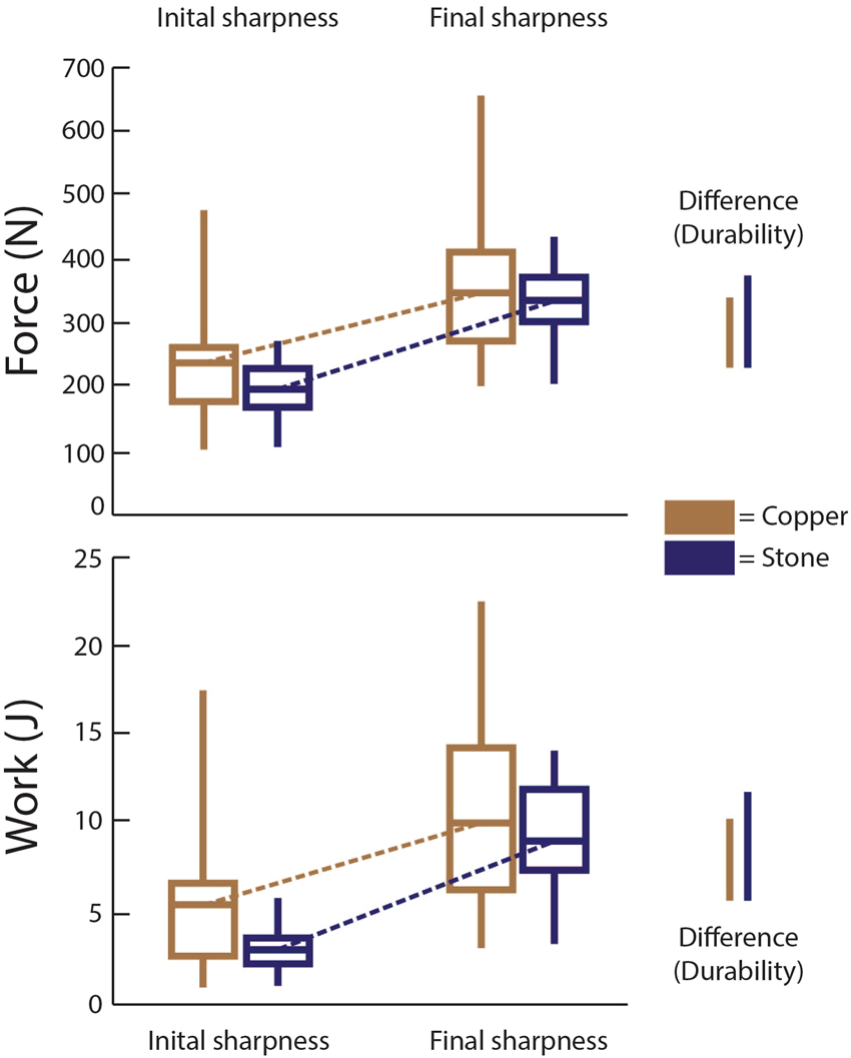
The force (N) and work (J) necessary for copper (brown) and stone (dark blue) blades to cut through a substrate. Stone blades are significantly sharper than copper ones initially, and after blunting there is no difference. Copper is more durable given it loses less sharpness.

The results show stone knives are sharper than copper ones. The results for force (*U* = 312.00, *p* = 0.041, *r* = 0.26) show that the copper blades ($\bar{x}$ = 237.90 N) required significantly more force to initiate and complete a cut than did the stone blades ($\bar{x}$ = 193.59 N) (Fig. 1). Likewise, the results for work (*U* = 257.00, *p* = 0.004, *r* = 0.37) were highly significant and demonstrate that copper blades ($\bar{x}$ = 5.424 J) require much more energy expenditure than do stone blades ($\bar{x}$ = 2.939 J) to complete the initial cut (Fig. 1).

### Final sharpness

The results show no difference between stone (force: $\bar{x}$ = 334.39 N; work: 9.099 J) and copper (force: $\bar{x}$ = 347.55 N; work: 10.101 J) knives after each was blunted (Fig. 1). There was no significant difference between the two groups either in the amount of force (*U* = 426.00, *p* = 0.723) and the amount of work (*U* = 429.50, *p* = 0.762) needed to cut the substrate.

### Durability

Copper knives were more durable than the stone knives (Fig. 1). The copper knives showed an increase between initial and final sharpness of 109.65 N and 4.677 J, while the stone knives showed an increase between initial and final sharpness of 140.04 N and 6.16 J. The copper blades’ increase in force and work required to cut the substrate was significantly less than that of the stone knives (force: *U* = 296.00, *p* = 0.023, *r* = 0.29; work: *U* = 302.00, *p* = 0.029, *r* = 0.28).

## Discussion

The selection against metal in the evolution of human technology is a rare occurrence. Why would people select against what is widely perceived to be a ‘superior’ raw material – metal – and revert back to a seemingly ‘inferior’ one – stone? Yet, by 3000 B.P., Late Archaic foraging societies of the North American Upper Great Lakes transitioned away from the utilitarian copper tools they had been using for millennia^1,13–17^. While demographic and social factors likely played a role in this event^13–15,17,19,27^, the role of copper versus stone durability and sharpness has not previously been investigated – despite Binford’s^19^ now 50-year-old discussion and explicit calls for experimentation.

Our results demonstrated that North American copper knives are more durable than analogous ones made from stone, supporting Binford’s^19^ assumption. But stone knives are initially sharper, and after an equal number of blunting events, copper and stone knives possess the same sharpness. Thus, copper knives’ greater durability does not actually provide any advantage in terms of functional efficiency. A tool-user might as well receive the front-loaded advantage of stone knives’ initial sharpness knowing that the greater rate of stone sharpness loss over several blunting events will ultimately result in a stone knife of the same functional efficiency as a copper knife having undergone the same amount of blunting. It is important to emphasize that these results do not consider the energy required to produce copper or stone tools, with copper requiring substantially more^19^, further increasing the efficiency advantages of stone. Overall, these results are consistent with the hypothesis that the selection against metal utilitarian tools by North American Late Archaic foragers required multiple contributing factors: demography, social reasons, *and* functional efficiency. Unless all of these factors act in concordance, humans will select metals over stone – which is what we typically see in the global archaeological record – and metal tools will eventually predominate over, or entirely replace, stone ones. In other words, the Old Copper Culture technomic devolution was likely an accident of history.

Two broad questions warrant further consideration. First, was functional efficiency a predominate or minor contributor to the Old Copper Culture technomic devolution, and was this contribution in terms of absolute efficiency, that is functional efficiency independent of production costs, or in terms of overall net energy expenditure? Second, what is the comparative functional efficiency of stone and metal utilitarian implements in prehistoric contexts where metal predominates or replaces stone? To better understand these questions, a comprehensive, experimental program is needed that engages with a variety of analogous tool types made from both copper and stone, which records the energetics of producing each, and assesses efficiency while using them. Additionally, with respect to the second question, light will be thrown on the differential evolutionary success of metal technology in different parts of the prehistoric world via direct comparisons between New World and Old World copper in terms of their elemental and geochemical composition, methods of production, and resulting materials properties.

## Supplementary information

The exceptional abandonment of metal tools by North American hunter-gatherers, 3000 B.P.

Supplementary Dataset 1
