# Supplementary material for: The exceptional abandonment of metal tools by North American hunter-gatherers, 3000 B.P
Source: Sci Rep. 2019 Apr 8;9:5756. doi: 10.1038/s41598-019-42185-y (PMC6453894; doi:10.1038/s41598-019-42185-y)
Supplement: Supplementary file 1 — The exceptional abandonment of metal tools by North American hunter-gatherers, 3000 B.P. [file 41598_2019_42185_MOESM1_ESM.docx]

Supplementary Information for:

**The exceptional abandonment of metal tools by North American hunter-gatherers, 3000 B.P.**

Michelle R. Bebber

Alastair J.M. Key

Michael Fisch

Richard S. Meindl

Metin I. Eren

Michelle R. Bebber

Email:

[mbebber@kent.edu](mailto:mbebber@kent.edu)

**This file includes:**

Supplementary text

Figs. S1 to S5

Table S1

References for SI reference citations

**Other supplementary materials for this manuscript include the following:**

Dataset S1

Supplementary Information Text

**Copper knives.**

Old Copper Culture knives (**Fig. S1**) are finely crafted and represent one of the most commonly found copper tool types in North America. These implements were first typed by Wittry^1^, and are classified as Wittry Group II^1^. Knives are technically defined as flat blades, asymmetrical in planview, with one side being slightly to moderately curved^2^. The curved side is commonly beveled to function as the “working edge”. This curved lateral portion is easily identified, as it tends to have more visual weight being wider towards the handle and then tapering towards the tip. Some knives have a blunt, rounded tip, while others were shaped to a point, giving the appearance of an asymmetrical projectile point. The opposing non-working side may be flat and thicker, or in some instances it is curved and beveled as well. Wittry Type II-A are defined as having a straight back with curved blade, while Wittry Type II-B have a curved back^1,3^. Analysis of the working edge^4^ shows that there is much variation. Some of the knife-edges were work hardened while others were left in the softer annealed state. It is apparent that the edges had been hammered extensively during shaping and finishing. Likewise, extensive grinding was required to make a shape knife edge. Leader^4^ notes the presence of edge chipping and twisted tips on the knives, suggesting they had been used for robust activities such as splitting, chopping, or prying apart objects. Old Copper Culture knives have various types of proximal (hafting) ends. The most common haft types are rat tailed or socketed tangs, but some specimens have rounded tangs, flattened tangs, and on occasion, hooks. The knives from the Middle and Late Archaic represent the earliest instance of humans manipulating metal to creating an elongate, curved blade edge designed specifically for cutting tasks.

Despite occasional claims to the contrary, there is much evidence that Old Copper Culture knives were used in utilitarian contexts, and not just ritual or symbolic ones^1,3,4,5,6,7,8,9^.

**Extended descriptions of materials and methods.**

**Tool sharpness**. Sharpness is one of the key attributes affecting a tool’s cutting efficiency and refers to the ability of a blade to initiate a cut at low force and deformation^10,11^. Often in past archaeological studies, sharpness has been incorrectly inferred based on the edge angle of the blade^12,13,14,15^. However, ergonomic and engineering research shows that sharpness can be effectively measured using force properties^11^ or a combination of geometric and mechanical approaches^10^.

**Tool edge angle.** Edge angle is a key trait affecting tool cutting efficiency. Mechanical performance testing for modern metal blades has shown, to various extents, the role edge angle plays in overall cutting ability^16,17,18^. For archaeological applications designed to evaluate tool cutting efficiency using human actors^13,14,15,19,20,21^ stone tool edge angle was variably influential factor that was interrelated to other ergonomic factors and overall tool size. A recent study^10^, which tested the force requirements of standardized blades on a materials tester, demonstrated that more obtuse edge angles require significantly more force, greater displacement, and more energy (work) to initiate and complete a cut.

**Cutting efficiency.** The primary purpose of a knife is to cut, slice, split, or otherwise deform material^22^. Within the realm of mechanical engineering, “cutting” is defined the initiation of a fracture which then causes the material to separate to some extent^16^. In an archaeological setting, the type of knives found in Old Copper Culture contexts would mostly likely have been used in a slicing motion. Slicing is defined as “the severance of two potions of material via the lineal movement of an edged tool across a material concurrent with the perpendicular pressing of the tool into the material. No waste material is created and separation is caused by material severance”^22^.

The mechanical properties involved in cutting can be quantified by measuring the force (N) and energy (J) necessary to complete a cut^16^. Work (J) is most closely tied to energy efficiency, as its measure allows us to evaluate the amount of energy expenditure required from the tool user during a task. Cutting implements experience bluntness due to wear and loss of material at the blade tip. The rate at which the blade edge begins to wear is largely dependent on the type of material being cut, the type of material of which the blade is made, and the conditions of the cutting action^16^. In real world settings, blades may dull due to a variety of reasons. For this study the goal was to mimic a wear inducing scenario that likely occurred in the past—contact with mammalian cortical bone during butchery.

**Investigating prehistoric copper cutting efficiency.** We set out to accurately characterize the mechanical principles that determine cutting efficiency for copper implements. Little comparative data exists for engineering principles applied to copper blades as it is not often, if ever, used in it pure form to make utilitarian implements as it was during the North American Copper Culture. It is most commonly alloyed with tin to make bronze, or with zinc to make brass^23,24^.

Key^22^ illustrates the ways in which mechanical principles from engineering and ergonomic sciences can be applied to archaeological lithics to further our understanding of tool form variation and past human behavior. Here we expand this body of research to analyze the energy differentials in task performance between copper blades and stone blades.

**Replica copper knife blade production.** We procured copper from the Adventure Mining Company (Greenland, Michigan), which is located in the same mining area that would have been used in ancient times, the Keweenaw Peninsula.

Earlier studies have demonstrated via experimentation^25^ and microscopic analysis^26,27^ that Old Copper Culture craftspeople likely used various production methods to manufacture and maintain their copper implements, including cold hammering, annealing, hot hammering, and grinding. Copper from the Lake Superior deposits is very pure, over 95% Cu, and thus could be shaped into tools without the use of smelting technology^2^.As such, forging, which involves repetitive compressing of the copper via hammering, was the primary smithing technique used by native North American metallurgists^25^.

The copper blade manufacture began with heating the raw copper nugget. After the initial heating and hammering, which functioned to remove the surface patination and accessory minerals, the copper was subjected to an alternating series of hot hammering, cold hammering, and annealing^2,25^. The length of production varied with each nugget due to the natural characteristics of the specimen and the amount of impurities contained within each copper piece.

The copper was shaped into an elongate blade form (**Fig. S2a**) which measured approximately 15 cm long and 2 cm wide. The blade was relatively flat and blunt on the top side, but was hammered so that it tapered toward the blade edge. Once the elongate blade form was complete, it was then cut via hammering on the sharp corner of an anvil into smaller blade sections measuring 2-3 cm in length and ~2 cm in width (**Fig. S2b**). This blade design was chosen to correspond to the length and width of stone flakes used in a previous study^10^. The thickness of the copper blade was designed to be consistent with Old Copper Culture archaeological specimens. Copper knife blades were analyzed from several museums including the Milwaukee Public Museum, The Chicago Field Museum, and the Canadian Museum of History (**Fig. S1**).

Once cut, the blades were then finished by pounding in the hot hammered state. After the blades cooled, they were polished by repetitive rubbing against wet sandstone, which functioned to remove the black surface residue and to further taper the blade edge. The blade edge was finalized by filing horizontally on a standard metal file and then rubbing each side of the blade edge on a whetstone 25 strokes (**Fig. S2c**).

**Replica stone flake production.** Stone flakes were produced using direct hard hammer percussion on Keokuk chert.

**Edge angle analysis.** A study on edge angle calculations^28^ demonstrate that both the graph method and the clay method are highly inaccurate with interobserver error up to 51%. Therefore, in this study edge angles for both stone and copper blade edges were calculated using the caliper method^28^, which has an interobserver error of only 2.4%. In order to control for this variable in our sharpness and durability tests, we selected each copper blade specimen edge angle (n=30) to correspond to that of a stone blade edge angle (n=30).

Copper blade edge angles were calculated first. To do so, calipers were set to 1 mm and edge widths were taken at three points 0 mm, 5 mm and 10 mm along a designated 1 cm segment of the blade edge. Edge angles were calculated independently by two of the authors (M.R.B. and A.J.M.K.) and then averaged (**Table S1**). Once the copper blade edge angles were established, stone blades were selected with corresponding edge angles. All edge angle pairs were within 1 degree of each other. Copper edge angles had a range of 27.3° (27.6° to 54.9°) and stone edge angles had a range of 27.8° (28.3° to 56.1°). The coefficient of variation for copper edge angles was 15.7 and 15.8 for stone edge angles (**Table S1**). There were no significant differences between the samples in either central tendency or dispersion (*U* = 447.0, *p* = .965 and *F*_(1,30)_ = .000, *p* = .986). Raw edge angle data is available in Dataset S1.

**Knife blunting procedure.** First, each of the copper or stone blades was tested for initial sharpness in the “fresh” condition where the blade edge has not been in contact with any substrate nor exposed to any damage. After the initial cut, and between each of the subsequent five cutting events, both types of blade were subjected to a controlled blunting event and then retested for sharpness using the established parameters. It is noted that even in modern meat processing using industrial steel blades, contact with bone during butchery will cause damage to the knife-edge^16^. As such, our procedure of five blunting events is a quite robust one. In order to mimic the type of blunting that may have occurred during the North American Archaic, our blades were used to cut a piece of bone—a distal end of a tibia from a white tailed deer (*Odocoileus virginianus*). Deer is the most widely represented subsistence fauna during the Archaic and subsequent periods, and thus deer bone represents a valid blunting mechanism. To perform the blunting, each blade was held perpendicular to the bone surface and then pulled in a slow controlled manner across the bond in order to maintain consistent pressure and angle during the blunting event.

**Experiments.** Our experiment here follows closely the procedures described in a previous experiment^10^ and some of what follows in this section has been reproduced from that study with appropriate and specific modifications particular to the present research question.

The PVC tubing substrate was secured to a custom made grip (**Fig. S3**). The grip setup consisted of two wooden blocks, spaced 40 mm apart, and mounted to a wood base. For each test run, a new length PVC tubing was placed horizontally between the wooden blocks and secured in place by metal clamps. Textured rubber strips were used to hold the side of the PVC tubing that was in direct contact with the metal clamp. The rubber pieces functioned to both protect the tubing while under tension and also provided additional grip to prevent movement of the tubing during the test. This system allowed for consistent placement and tension of the tubing for each test.

Both the stone and copper blade samples were mounted in standardized wooden blocks measuring 4 x 2 x 12 cm, which were designed to fit in the crosshead. To mount each blade in the wood block, first appropriate sized notches (which varied with blade width) were cut into the wood support blocks. The blades were then carefully oriented and glued into place so that the cutting edge was parallel to the support block. To run each test, the wooden support block containing the blade was mounted into the load bearing crosshead using Instron Universal Materials Tester (Model 5967) screw action grips.

**Experimental Data Collection.** An Instron Universal Materials Tester (Model 5967) universal testing machine was used to perform compressive tests (**Fig. S4**). This type of test continuously measures applied load (stress) and the associated displacement response (strain). In this study, the stress was introduced and controlled entirely by the Instron testing apparatus. A crosshead displacement rate of 0.2 mm/per minute was used in configuration with the substrate base. The test involves placing the cut substrate mounted in the grip base onto the base below the crosshead. The cutting edge of the blades was oriented so that it was perpendicular to the substrate at all times. The crosshead was then carefully lowered so the predesignated midpoint of the blade tip—the point at which the edge angle was calculated—was in contact with the surface of the PVC tubing. At this point, prior to beginning the test data collection, the force (N) was balanced and the displacement (mm) reset to zero.

As the test proceeded, the amount of load was continuously measured as the sample underwent displacement due to the applied force. The Instron Bluehill Universal software (Version 4.04) connected to the testing apparatus continuously collected measurements throughout the test duration. Deflection curves were generated for each sample (**Fig. S5**). The x-axis shows the amount of displacement (mm) and the y-axis shows the amount of applied force (N). The relationships between these parameters are used to calculate measures that reveal the blades sharpness and response to blunting. The load and displacement data were used to calculate two key variables: *max load* and *work.* Max load refers to the maximum amount of load applied during the test. Load was measured in Newtons/meter. This measure provides an assessment of initial force required to introduce a fracture by measuring the maximum of amount of load withstood by the substrate prior to cut initiation. The *work* value, measured in joules, illustrates the amount of energy needed to complete the cut. Work is calculated from the area under the force displacement curve from where the sample begins deformation, through initial fracture, and continuing until complete cutting of the material occurs. Six different sharpness conditions were recorded in this study. The initial “fresh” values were recorded for each test sample, followed by five blunted conditions. All raw data can be found in Dataset S1.

**Additional analysis.**

**Amount of blunting.** How much blunting actually occurred between initial and final sharpness? We can assess this by comparing initial to final sharpness within each material type. An analysis of copper blade data shows that there is a significant difference between the initial sharpness and final sharpness with large effect values for both amount of force (N) (*U* = 166.00, *p* < .000, *r* = .54) and work (J) (*U* = 186.00, *p* < .000, *r* = .50) necessary to cut the substrate. Similarly, an analysis of stone blade data also shows there is a significant difference between initial and final sharpness with large effect values for both amount of force (N) (*U* = 24.00, *p* < .000, *r* = .81) and work (J) (*U* = 20.00, *p* < .000, *r* = .82) necessary to cut the substrate.

These results confirm that the blunting method used herein—controlled contact with animal bone—does in fact cause reduced cutting efficiency for both copper and stone blades. Raw data are available in Dataset S1.


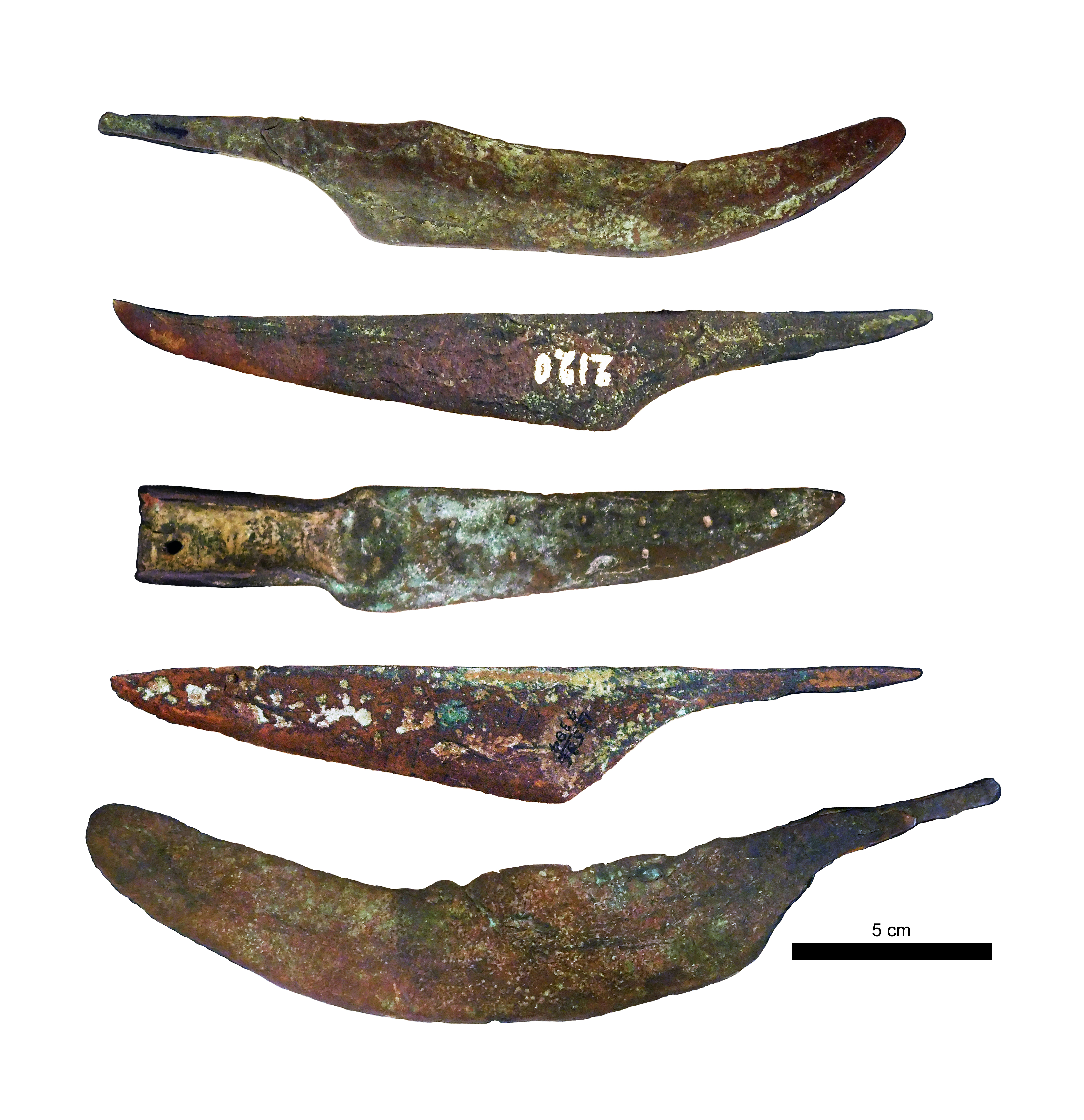


**Fig. S1**. Archaeological examples of Old Copper Culture copper knives curated at the Milwaukee Public Museum. Photographed by M.R.B. July 18th, 2017.


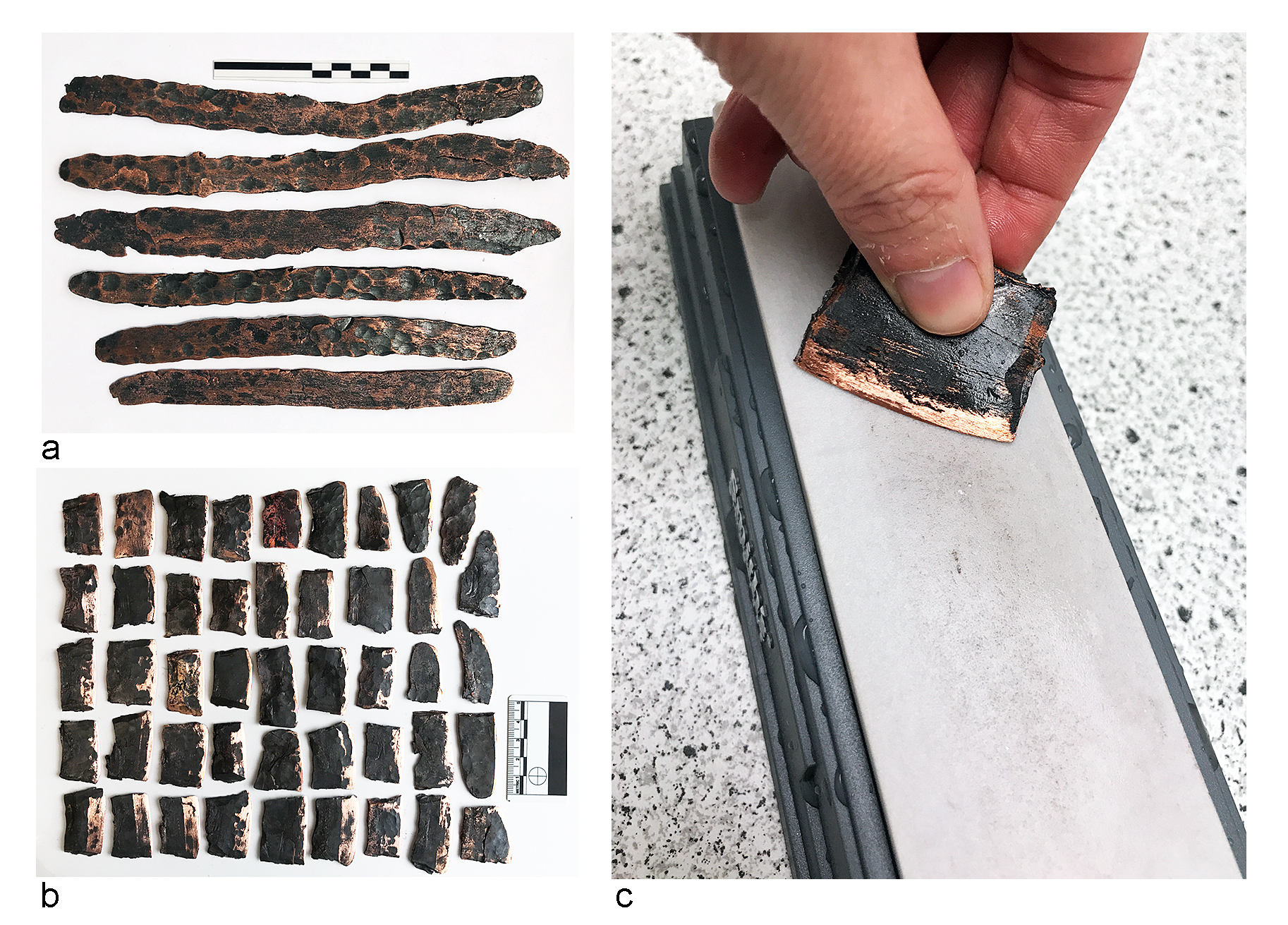


Fig. S2. Copper blade production showing stages (a) elongate copper ingots, (b) tapered blades cut into segments, and (c) final blade sharpening on whetstone.


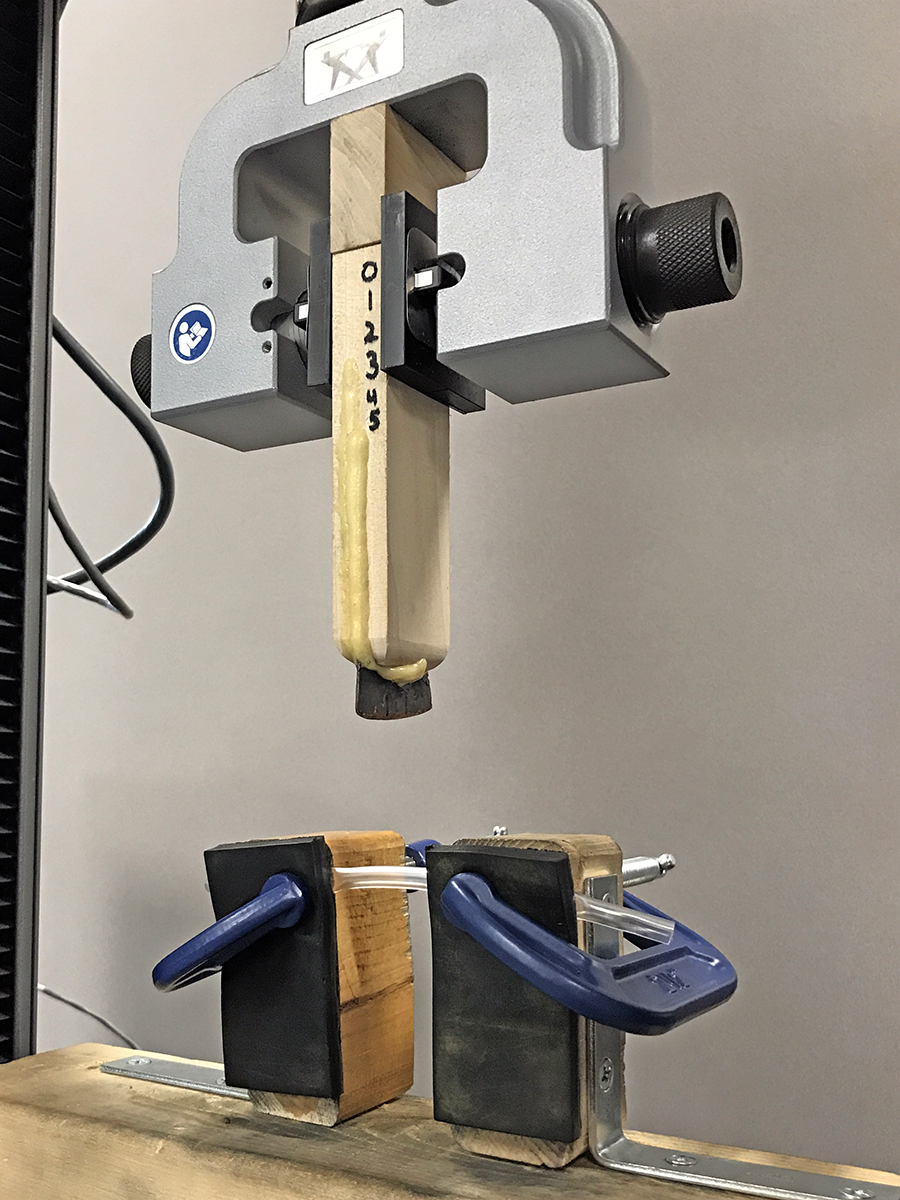


Fig. S3. Testing apparatus showing copper blade, mounted in wood block, held by Instron screw action grips being lowered towards the PVC cutting substrate.


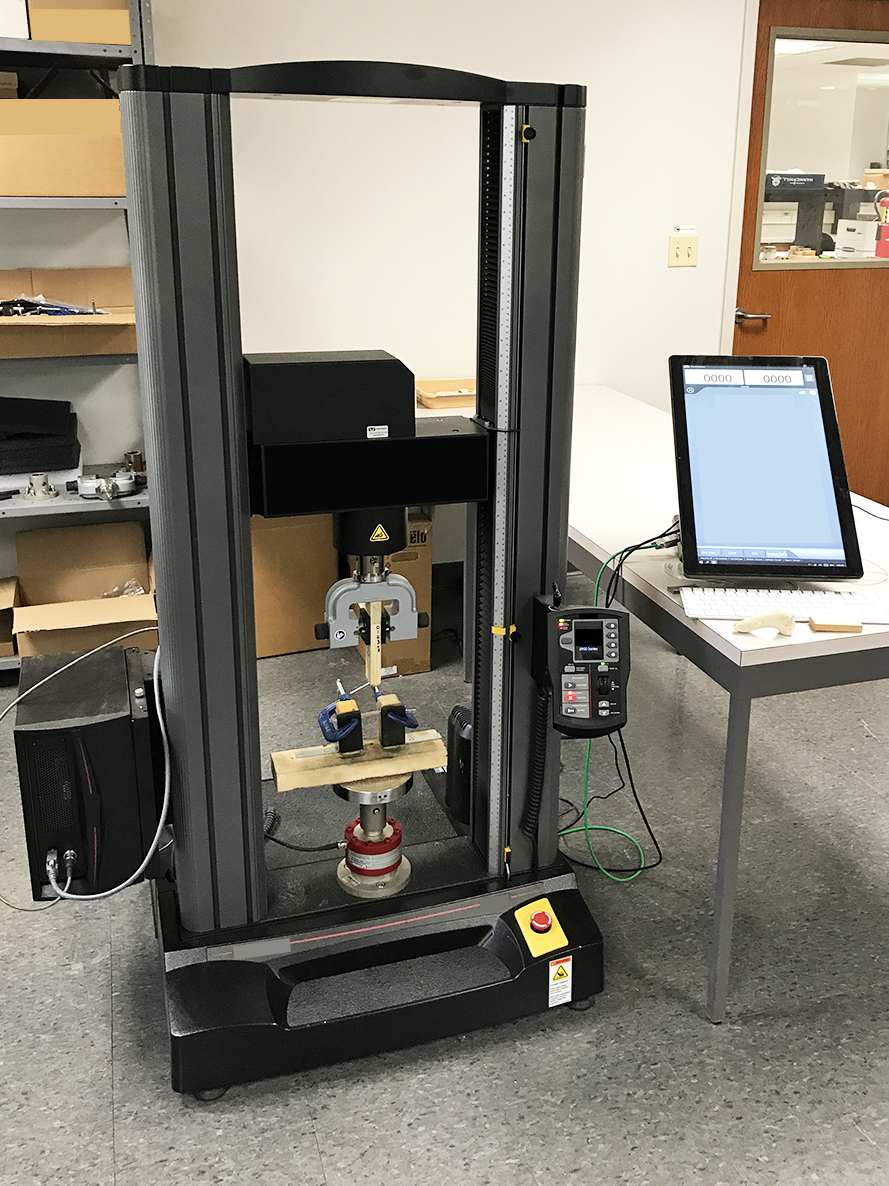


Fig. S4. Instron Universal Materials Tester (Model 5967) was used to perform compressive tests and to collect all data.


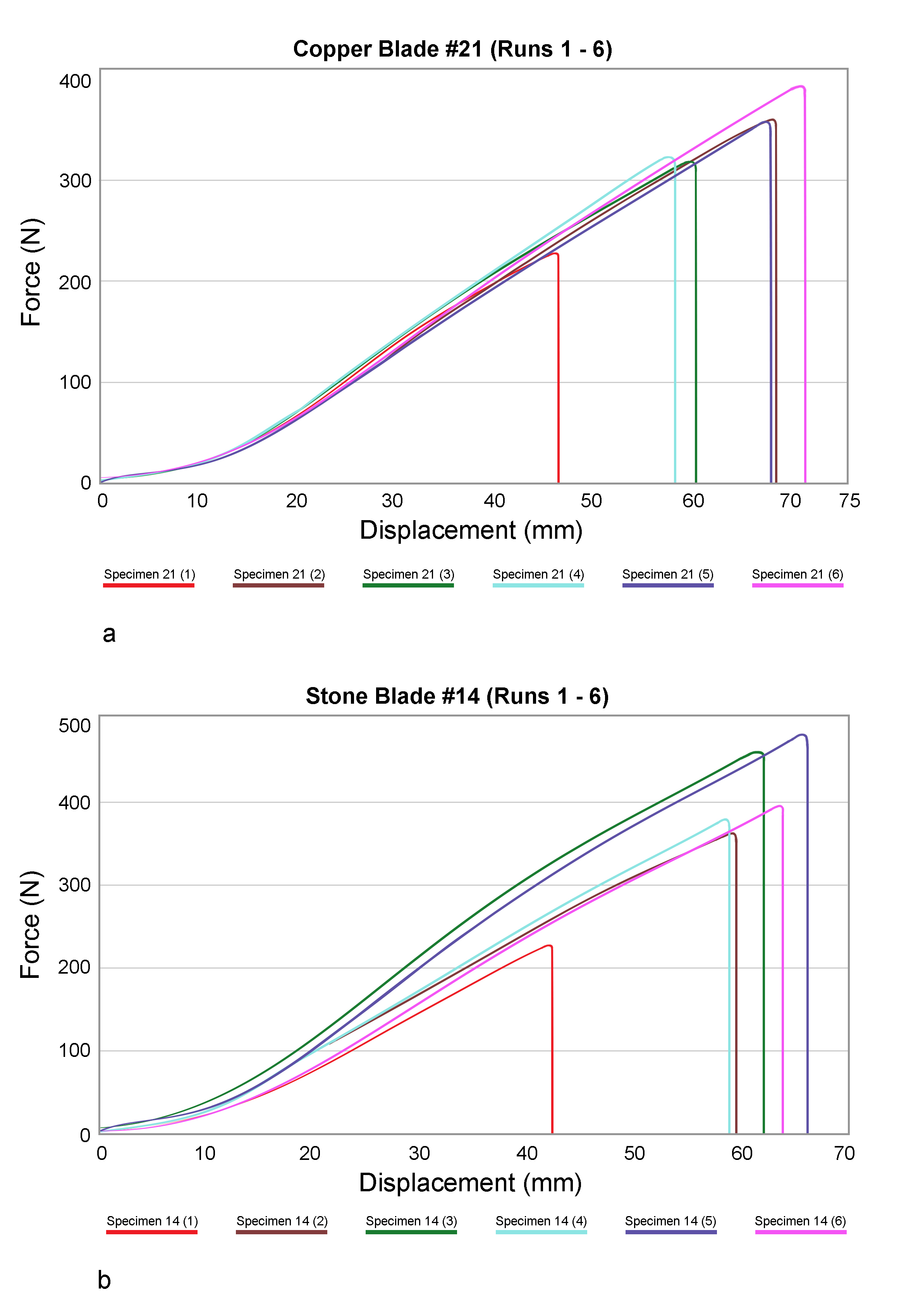
Fig. S5. Load displacement curves depicting a typical test for (a) copper blades and (b) stone blades. Data for each blade has been plotted for conditions one (1) though six (6).

**Table S1.** Blade edge angle in degrees °

|  | Copper | Stone |
| --- | --- | --- |
| Mean | 44.1 | 44.0 |
| StDev | 6.9 | 7.0 |
| CoVar | 15.7 | 15.8 |
| Min | 27.6 | 28.3 |
| Max | 54.9 | 56.1 |
| Range | 27.3 | 27.8 |

**References**

1. Wittry W (1951) A preliminary study of the old copper complex. *Wisconsin Archeol* 32:1–18.

2. Martin S (1999) Wonderful Power: the Story of Ancient Copper Working in the Lake

Superior Basin. Great Lakes Books Series. Wayne State University Press.

3. Penman J (1977). The old copper culture: an analysis of old copper artifacts. *Wisconsin Archeol*, 58:3–23.

4. Leader J-M (1988) *Technological continuities and specialization in prehistoric metalwork in the eastern United States.* Ph.D. dissertation. Department of Anthropology, University of Florida, Gainesville.

5. Gibbon G (1998) Old copper in Minnesota: a review. Plains Anthropol 43:27–50.

6. McHugh W-P (1973) “New Archaeology" and the Old Copper Culture. *Wisconsin Archeol* 54:70–83.

7. Pleger T (1998) Social Complexity, Trade, and Subsistence during the Archaic/

Woodland Transition in the Western Great Lakes (4000-400 B.C.): a Diachronie Study of Copper Using Cultures at the Oconto and Riverside Cemeteries. Ph.D. dissertation. Department of Anthropology, University of Wisconsin-Madison. University Microfilms International, Ann Arbor, Michigan.

8. Pleger T (2000) Old copper and red ocher social complexity. Midcont J Archaeol 25:169–190.

9. Pleger T, Stoltman J (2009) The Archaic Tradition in Wisconsin. Archaic Societies: Diversity and Complexity across the Midcontinent, eds Emerson T, McElrath D, Fortier A (SUNY Press, Albany, NY), pp 697–724.

10. Key A, Fisch M, Eren, M (2018) Early stage blunting causes rapid reductions in stone tool performance. *J Archaeol Sci* 91:1-11.

11. Schuldt, S., Arnold, G., Roschy, J., Schneider, Y. and Rohm, H., 2013. Defined abrasion procedures for cutting blades and comparative mechanical and geometrical wear characterization. *Wear*, *300*(1-2), pp.38-43.

12. Jobson R-W (1986) Stone tool morphology and rabbit butchering. *Lithic Technology*, *15*(1), 9-20.

13. Key A-J, Lycett S-J (2015) Edge angle as a variably influential factor in flake cutting efficiency: an experimental investigation of its relationship with tool size and loading. *Archaeometry*, *57*(5), 911-927.

14. Key A-J, Proffitt T, Stefani E, Lycett S-J (2016) Looking at handaxes from another angle: Assessing the ergonomic and functional importance of edge form in Acheulean bifaces. *Journal of Anthropological Archaeology*, *44*, 43-55.

15. Merritt S-R (2016) Cut mark cluster geometry and equifinality in replicated Early Stone Age butchery. *International Journal of Osteoarchaeology*, 26:585–59.

16. Atkins T (2009) *The science and engineering of cutting: the mechanics and processes of separating, scratching and puncturing biomaterials, metals and non-metals*. Butterworth-Heinemann.

17. McCarthy, C. T., Hussey, M., & Gilchrist, M. D. (2007). On the sharpness of straight edge blades in cutting soft solids: Part I–indentation experiments. *Engineering Fracture Mechanics*, 74:2205–2224.

18. McGorry R-W, Dowd P-C, Dempsey P-G (2005) The effect of blade finish and blade angle on forces used in meat cutting operation. *Appl. Ergon.*, 36(1), pp. 71-77.

19. Collins S (2008) Experimental investigations into edge performance and its implications for stone artefact reduction modelling. *J Archaeol Sci* 35:2164–2170.

20. Jones P-R (1980) Experimental butchery with modern stone tools and its relevance for Palaeolithic archaeology. *World Archaeology* 12:153–165.

21. McCall G-S (2005) An experimental examination of the potential function of Early Stone Age tool technology and implications for subsistence behavior. *Lithic Technology* 30:29–43.

22. Key A-J (2016) Integrating mechanical and ergonomic research within functional and morphological analyses of lithic cutting technology: key principles and future experimental directions. *Ethnoarchaeology* 8:69–89.

23. Davis J-R (2008) *ASM Specialty Handbook: Copper and Copper Alloys* (ASM International, Metals Park).

24. Notis M-R (2014) Metals. *Archaeometallurgy in global perspective: methods and syntheses*, eds Roberts B-W, Thornton C-P (Springer Science & Business Media, Heidelberg), pp. 47–66.

25. LaRonge M (2001) An experimental analysis of Great Lakes Archaic copper smithing. *N Am Archaeol* 22:371–385.

26. Fregni G (2009) A study of the manufacture of copper spearheads in the Old Copper Complex. *Minnesota Archaeologist* 67:121–130.

27. Vernon W (1990) New archaeometallurgical perspectives on the old copper industry of North America. Geological Society of America Centennial Special, vol. 4. pp. 499–512.

28. Dibble H-L, Bernard M-C (1980) A comparative study of basic edge angle measurement techniques. *Am Antiquity*, *45*:857–865.
